# Challenges in Diagnosing and Managing Hepatitis E in Transplant Recipients: A Case Report

**DOI:** 10.1155/crit/3747751

**Published:** 2026-02-12

**Authors:** Ana Cristina Martins, Gonçalo Pimenta, André Mascarenhas, Rita Veríssimo, Sara Querido, André Weigert

**Affiliations:** ^1^ Nephrology Department, Unidade Local de Saúde de Lisboa Ocidental, Lisbon, Portugal; ^2^ Gastroenterology Department, Unidade Local de Saúde de Lisboa Ocidental, Lisbon, Portugal

**Keywords:** Hepatitis E, immunosuppression, kidney transplant, liver cytolysis

## Abstract

Hepatitis E virus (HEV) is a common but often underdiagnosed cause of hepatitis in both the general population and transplant patients. Diagnosis is usually made by detecting IgM anti‐HEV antibodies, with immunosuppressed individuals being at higher risk for chronic infection. We present the case of a 56‐year‐old man who developed asymptomatic liver cytolysis 4 months after a kidney transplant, with no significant physical findings or relevant epidemiological history. Abdominal ultrasound revealed hepatic steatosis. Drug‐induced liver injury was suspected, prompting the discontinuation of potential causative agents. However, liver enzymes worsened asymptomatically after 2 weeks, prompting further medication adjustments. Testing for Hepatitis B and C, as well as IgM for *Toxoplasma*, was negative. Serologies for herpes simplex and varicella zoster were positive for IgG and negative for IgM, and CMV viral load was undetectable. HEV serologies were negative for IgG and IgM. A liver biopsy revealed nonspecific acute hepatitis without fibrosis. The positive HEV plasma viral load was suggestive of Hepatitis E. As the patient was asymptomatic with no further analytical worsening, targeted treatment was not initiated. The patient achieved spontaneous viral clearance in less than 12 weeks, despite his immunocompromised condition. This case report highlights the diagnostic challenges of HEV hepatitis in immunosuppressed patients, as detection may rely solely on viral load testing due to potential serological negativity. This case also highlights that despite the patient′s immunosuppressed condition, viral clearance is possible without the need for targeted antiviral therapy.

## 1. Introduction

Hepatitis E virus (HEV) is a leading cause of acute viral hepatitis worldwide, with immunocompromised patients being at high risk of developing chronic infection [1]. Although initially considered a disease that would only affect travelers and populations within developing nations, its prevalence is increasing in the West [1, 2]. Certain behaviors present a risk factor for HEV infection, such as consumption of raw meat, exposure to soil, blood transfusions, traveling to endemic areas, contacting dogs, and living in rural areas [1]. In industrialized countries, HEV Genotype 3 is more prevalent and typically transmits zoonotically through undercooked meat consumption, unlike Genotypes 1 and 2 which spread via the fecal–oral route [3, 4].

The clinical presentation is heterogeneous, ranging from asymptomatic cases to severe manifestations including fatigue, abdominal pain, dark urine, and jaundice. HEV infection is diagnosed either indirectly by detecting anti‐HEV antibodies or directly by identifying HEV RNA using quantitative reverse transcription polymerase chain reaction (RT‐PCR) in a biological sample (serum, plasma, or stool) [4]. In particular, in immunosuppressed patients, virus detection by RT‐PCR should always be attempted, as anti‐HEV‐IgM was not positive in nearly one‐fourth in a recent study [2]. Management initially involves waiting for spontaneous clearance of the virus; in transplant recipients or other immunocompromised patients, the first step is to attempt a reduction in immunosuppression. If this fails to clear the infection within 12 weeks, ribavirin is initiated, although this approach increases the risk of graft rejection [3, 5].

We report the case of a HEV infection lacking detectable anti‐HEV IgG and IgM in a renal transplant recipient.

## 2. Case Presentation

A 56‐year‐old man from Venezuela, who had been a kidney transplant recipient for 4 months, had received basiliximab as induction immunosuppression and tacrolimus, mycophenolate mofetil, and prednisolone as maintenance immunosuppression. During a follow‐up consultation, asymptomatic liver cytolysis was detected (ALT 775 U/L, AST 483 U/L, GGT 113 U/L, and ALP 181 U/L), with no abnormalities found on physical examination and no relevant epidemiological context, including alcohol consumption. Abdominal ultrasound only revealed hepatic steatosis. In order to exclude drug‐induced liver injury (DILI), all potential causative agents (trimethoprim/sulfamethoxazole, valganciclovir, and pantoprazole) were discontinued. Analytical asymptomatic worsening was observed after 2 weeks (ALT 1080 U/L, AST 686 U/L, GGT 152 U/L, and ALP 214 U/L), leading to further medication adjustments (discontinuation of atovaquone and conversion from tacrolimus to cyclosporine). Immunological studies, serologies, and viral loads for Hepatitis B and C were negative, as well as IgM for *Toxoplasma gondii*. Serological testing for Hepatitis A showed positive anti‐HAV IgG and negative anti‐HAV IgM. EBV serologies revealed positive anti‐EBNA IgG and anti‐VCA IgG, with negative anti‐VCA IgM. Serologies for herpes simplex and varicella zoster were positive for IgG and negative for IgM. CMV plasma viral load was undetectable. HEV serologies were negative for IgG and IgM, with the plasma viral load result pending.

A liver biopsy revealed moderate acute hepatitis with a preserved trabecular architecture, moderate lymphocytic inflammation, interface and scattered necrosis, and cholestasis. No significant fibrosis, iron deposition, or steatosis were observed. These findings were consistent with acute hepatitis, potentially of viral or toxic etiology. Subsequently, the HEV plasma viral load result came back positive, establishing the diagnosis of Hepatitis E. Since the patient was asymptomatic and showed no analytical worsening, targeted treatment was not initiated. However, immunosuppression was altered (tacrolimus was replaced by cyclosporin), and the patient remained under surveillance. The patient achieved spontaneous viral clearance and resolution of liver test abnormalities in less than 8 weeks, despite his immunocompromised condition. Immunosuppression therapy was adjusted, switching from cyclosporine back to tacrolimus. Recommendations were reinforced to prevent zoonotic infections and those transmitted via the fecal–oral route. Three years after the episode, the patient remained asymptomatic with no recurrence of Hepatitis E and had adequate kidney function, as indicated by a creatinine level of 1.54 mg/dL and an estimated glomerular filtration rate of 49 mL/min. The patient continues to be treated with tacrolimus, with trough levels maintained between 5 and 7 ng/mL. On follow‐up, the patient tested positive for both anti‐HEV IgG and IgM antibodies, indicating ongoing serologic response.

## 3. Discussion

The patient′s clinical presentation was particularly notable for its asymptomatic nature despite marked liver enzyme abnormalities. Although posttransplant monitoring revealed a significant elevation in liver transaminases, the patient did not exhibit any typical symptoms associated with hepatitis, such as abdominal pain, jaundice, or fatigue. This absence of symptoms highlights one of the challenges of diagnosing Hepatitis E in immunocompromised patients, where liver enzyme abnormalities may be the only clue to the underlying infection. This observation is supported by a recent cohort study, which found that 74% of immunocompromised patients with Hepatitis E were completely asymptomatic [3].

In addition to being asymptomatic, this patient presented another factor that further complicated the diagnosis, which was the initial negative result for anti‐HEV‐IgM on ELISA, a standard marker used for detecting acute Hepatitis E [1]. The negative serology for anti‐HEV‐IgM, combined with the lack of clinical symptoms, initially obscured the diagnosis. This case underscores a critical difficulty in diagnosing Hepatitis E in immunocompromised patients, where serological tests may not always be reliable [3]. The definitive diagnosis was established through positive HEV viral load testing, which highlighted the presence of the virus despite serological negativity.

Although immunosuppressed patients with Hepatitis E often struggle to clear the infection due to their compromised immune systems and the use of immunosuppressive drugs like tacrolimus, which increase the risk of chronic infection, this patient cleared the viral load within 12 weeks and did not require pharmacological intervention [5]. A transient rise in HEV viral load that spontaneously resolves within a few weeks in a kidney transplant recipient can be explained by several possible mechanisms. First, fluctuations in immunosuppression, such as dose adjustments, temporary increases in immunosuppressive therapy, or treatment for rejection, may transiently impair antiviral immune control, allowing short‐lived viral replication that subsequently resolves once immune function stabilizes or immunosuppression is reduced. Second, host immune response dynamics may account for a brief period of detectable viremia before an effective immune response is mounted, especially in patients with partial immune competence or after immunosuppression tapering. Third, viral quasispecies shifts within the circulating HEV population may produce transient increases in replication efficiency, followed by rapid containment by the immune system [6]. Finally, intercurrent illness or coinfection can temporarily weaken immune surveillance and permit short‐lived increases in HEV replication, with spontaneous resolution as the underlying condition improves [7].

The decision to initiate ribavirin therapy was carefully considered, as per current recommendations for managing HEV infection in immunosuppressed patients. Current guidelines recommend initiating ribavirin in immunocompromised individuals with persistent HEV infection beyond 3 months or in those showing clinical or biochemical deterioration, especially when immunosuppression cannot be sufficiently reduced [8]. However, since the patient exhibited spontaneous viral clearance within a few weeks, with normalization of liver enzymes and no clinical symptoms, pharmacological treatment was deemed unnecessary. The rapid resolution of the infection in this case highlights the variability in disease progression and suggests that, even in immunocompromised individuals, spontaneous resolution of Hepatitis E is possible, though not the norm. Persistent infection requires the initiation of ribavirin therapy and a reduction in immunosuppressive treatment, thereby elevating the risk of graft rejection [3].

Although the presence of HEV RNA in plasma is considered the diagnostic gold standard for acute infection, particularly in immunosuppressed patients, the absence of detectable anti‐HEV IgG and IgM, combined with the absence of serial RNA testing during the acute phase, limits the diagnostic certainty in this case. According to current guidelines, chronic HEV infection is defined by the persistence of detectable HEV RNA for more than 3 months, which was not observed here [8, 9]. Importantly, follow‐up testing demonstrated spontaneous viral clearance within 8 weeks, reinforcing the likelihood of a self‐limited and benign disease course, even in the setting of immunosuppression. Moreover, the possibility of DILI cannot be definitively excluded, particularly given the temporal association between liver enzyme fluctuations and sequential medication adjustments. Although liver histology was consistent with acute hepatitis of either viral or toxic etiology, it lacked specific features to distinguish between the two [10]. The rapid and spontaneous resolution of HEV RNA in an immunocompromised patient is atypical but has been reported in selected cases [11]. Therefore, while the detection of HEV RNA supports the diagnosis of acute HEV infection, it should be interpreted with caution in this clinical context, and this case should be regarded as a probable, rather than definitive, HEV infection.

## 4. Conclusions

This case highlights the diagnostic complexity of HEV infection in immunocompromised transplant recipients, particularly when serologies are negative and only a single HEV RNA positive determination is available. Although the detection of viral RNA and the favorable clinical course support a probable diagnosis of HEV infection, alternative diagnoses such as DILI cannot be definitively excluded. Nonetheless, the spontaneous resolution of liver enzyme abnormalities and clearance of HEV RNA within 8 weeks suggest that, in selected cases, a conservative management approach may be appropriate. This report also underscores the importance of nucleic acid amplification testing in transplant recipients with unexplained hepatitis, even when serologies are negative.

## Consent

Patient consent was obtained.

## Conflicts of Interest

The authors declare no conflicts of interest.

## Funding

No funding was received for this manuscript.

## Data Availability

Data sharing is not applicable to this article as no datasets were generated or analyzed during the current study.

## References

[1] Li P. , Liu J. , Li Y. , Su J. , Ma Z. , Bramer W. M. , Cao W. , de Man R. A. , Peppelenbosch M. P. , and Pan Q. , The Global Epidemiology of Hepatitis E Virus Infection: A Systematic Review and Meta-Analysis, Liver International. (2020) 40, no. 7, 1516–1528, 10.1111/liv.14468, 32281721. 32281721 PMC7384095

[2] Chauhan A. , Webb G. , and Ferguson J. , Clinical Presentations of Hepatitis E: A Clinical Review With Representative Case Histories, Clinics and Research in Hepatology and Gastroenterology. (2019) 43, no. 6, 649–657, 10.1016/j.clinre.2019.01.005, 2-s2.0-85061871664, 30808575. 30808575 PMC6864596

[3] Kupke P. , Kupke M. , Borgmann S. , Kandulski A. , Hitzenbichler F. , Menzel J. , Geissler E. K. , Schlitt H. J. , Wenzel J. J. , and Werner J. M. , Hepatitis E Virus Infection in Immunosuppressed Patients and Its Clinical Manifestations, Digestive and Liver Disease. (2025) 57, no. 2, 378–384, 10.1016/j.dld.2024.06.020, 38997847. 38997847

[4] Donnelly M. C. , Scobie L. , Crossan C. L. , Dalton H. , Hayes P. C. , and Simpson K. J. , Review Article: Hepatitis E-a Concise Review of Virology, Epidemiology, Clinical Presentation and Therapy, Alimentary Pharmacology & Therapeutics. (2017) 46, no. 2, 126–141, 10.1111/apt.14109, 2-s2.0-85018670619, 28449246. 28449246

[5] Nimgaonkar I. , Ding Q. , Schwartz R. E. , and Ploss A. , Hepatitis E Virus: Advances and Challenges, Nature Reviews Gastroenterology & Hepatology. (2018) 15, no. 2, 96–110, 10.1038/nrgastro.2017.150, 2-s2.0-85045709292, 29162935. 29162935 PMC11329273

[6] Hoofnagle J. H. , Nelson K. E. , and Purcell R. H. , Hepatitis E, The New England Journal of Medicine. (2012) 367, no. 13, 1237–1244, 10.1056/NEJMra1204512, 2-s2.0-84866763098, 23013075. 23013075

[7] Lhomme S. , Abravanel F. , Dubois M. , Sandres-Saune K. , Rostaing L. , Kamar N. , and Izopet J. , Hepatitis E Virus Quasispecies and the Outcome of Acute Hepatitis E in Solid-Organ Transplant Patients, Journal of virology. (2012) 86, no. 18, 10006–10014, 10.1128/JVI.01003-12, 2-s2.0-84866173732, 22761386. 22761386 PMC3446597

[8] European Association for the Study of the Liver , EASL Clinical Practice Guidelines on Hepatitis E Virus Infection, Journal of Hepatology. (2018) 68, no. 6, 1256–1271, 10.1016/j.jhep.2018.03.005, 2-s2.0-85044575164, 29609832. 29609832

[9] Kamar N. , Izopet J. , Pavio N. , Aggarwal R. , Labrique A. , Wedemeyer H. , and Dalton H. R. , Hepatitis E Virus Infection, Nature Reviews Disease Primers. (2017) 3, no. 1, 10.1038/nrdp.2017.86, 2-s2.0-85034838890. 29154369

[10] Danan G. and Teschke R. , RUCAM in Drug and Herb Induced Liver Injury: The Update, International Journal of Molecular Sciences. (2016) 17, no. 1, 10.3390/ijms17010014, 2-s2.0-84951788964, 26712744. PMC4730261 26712744

[11] van der Valk M. , Zaaijer H. L. , and Kamar N. , Publisher′s Note, Liver International. (2017) 37, no. 5, 770–1499, 10.1111/liv.13445, 2-s2.0-85042728169.

